# Multifaceted approaches to promoting the growth potential in small-for-gestational-age infants and their clinical relevance

**DOI:** 10.12669/pjms.41.7.11010

**Published:** 2025-07

**Authors:** Lijuan Si, Jing Guo, Wenlong Du, Simeng Zhao, Jing Li

**Affiliations:** 1 Lijuan Si Department of Children’s Health Care, Baoding Maternal & Child Health Hospital, Baoding 071000, Hebei, China; 2 Jing Guo Department of Children’s Health Care, Baoding Maternal & Child Health Hospital, Baoding 071000, Hebei, China; 3 Wenlong Du Department of Children’s Health Care, Baoding Maternal & Child Health Hospital, Baoding 071000, Hebei, China; 4 Simeng Zhao Department of Paediatrics, Baoding Maternal & Child Health Hospital, Baoding 071000, Hebei, China; 5 Jing Li Department of Paediatrics, Baoding Maternal & Child Health Hospital, Baoding 071000, Hebei, China

**Keywords:** Catch-up Growth, Insulin Resistance, MDI, PDI, Small for Gestational Age, Z-score

## Abstract

**Objective:**

To investigate the multifaceted approaches to promoting growth potential in small-for-gestational-age (SGA) infants and their clinical relevance.

**Methods:**

This was a retrospective study. A total of 196 SGA infants who were born in the Department of Obstetrics at Baoding Maternal & Child Health Hospital from February 2020 to February 2024 were selected as the study subjects and were divided into the control group (n=98) and the catch-up group (n=98) according to different nursing approaches. Specifically, the clinical value was investigated using the various development index, and maternal anxiety and depression scores.

**Result:**

No significant differences were observed in body weight, height, WAZ, and HAZ between the 2 groups at 3 ~ 9 months of corrected age (P > 0.05), while the body weight, height, WAZ, and HAZ in the catch-up group were greater than those in the control group at 12 ~ 24 months of corrected age, with significant differences (P < 0.01). Meanwhile, at 24 months of correction age, PDI, MDI, FBG and Homa-IAI indexes in the catch-up group were higher than those in the control group, while FINS, Homa-IR, HBCI and anxiety and depression indexes were lower than those in the control group, with statistical significance (P<0.01).

**Conclusion:**

Early interventions in SGA infants with appropriate growth potential can achieve catch-up growth and prevent side effects of overnutrition-induced insulin resistance.

## INTRODUCTION

Small for Gestational Age (SGA) refers to newborns weighing less than the 10th percentile of the mean weight of those of the same sex and gestational age,^1^ and approximately 16% of live births worldwide fall into this category.^2^ The causes of SGA are complex and involve multiple factors, including maternal factors, placental and cord factors, genetic factors, fetal and environmental factors, and metabolic diseases.^3^,^4^ SGA infants who fail to achieve catch-up growth (CUG) may experience long-term cognitive and learning disabilities, severely impacting their normal lives while bringing a financial burden to their families and society.

In this study, multifaceted approaches to promoting the growth potential in SGA infants and their clinical relevance were investigated, aiming to improve the efficacy of SGA infants. At present, the domestic and foreign studies on SGA growth and development mainly focus on physical development or nervous system development, these studies are lack of systematic, there is a single problem. However, the quality of life of children is affected by a combination of many factors, and there are complex interactions between various interventions to promote growth and development. Therefore, multiple indexes, including body weight, height, motor and mental development, and insulin resistance were comprehensively assessed in this study, so as to thoroughly investigate the clinical value of approaches to promoting growth potential.

## METHODS

This was a retrospective study. A total of 196 premature infants born and hospitalized in the Department of Obstetrics at Baoding Maternal & Child Health Hospital from February 2020 to February 2024 were selected as the study subjects and divided into the control group (n=98) and catch-up group (n=98) according to whether or not early intervention nursing was performed, the control group receiving no early intervention nursing, while the catch-up group applied a variety of approaches to promoting growth potential for catch-up growth nursing. The demographic characteristics of premature infants in the 2 groups were not significantly different (P> 0.05), Table-I.

**Table-I:** Comparison of birth status of premature infants between the two groups.

Index	Catch-up group	Control group	χ^2^/t	P
Sex-M	46	40	0.746	0.388
Sex-F	52	58		
Gestational age	32.52±2.88	32.04±2.96	1.150	0.251
Body weight`(*χ̅*±*S*,g)	1673.57±164.00	1667.86±116.92	0.281	0.779
Height`(*χ̅*±*S*,cm)	43.76±2.07	43.30±1.55	1.763	0.080
5min Apgar score	7.84±1.33	7.74±1.07	0.533	0.595

### Ethical approval:

The study was approved by the Institutional Ethics Committee of Baoding Maternal and Child Health Hospital (No.: 2141ZF283; date: April 15, 2023), and written informed consent was obtained from all *participants’ guardians*

### Inclusion criteria:

- Gestational age of 28 - 37 weeks, birth weight below the 10th percentile of the mean weight of the same sex and gestational age.
- The infants and their family members voluntarily signed the relevant written documents and voluntarily received follow-up.
- The family members had the ability of normal communication and understanding and cooperation with the relevant treatment to ensure the accuracy and reliability of the study data.

### Exclusion criteria:

- Infants with congenital malformations, inherited metabolic diseases, or chromosomal diseases;
- Infants who were self-discharged, transferred, lost to follow-up, or died.

### Intervention Measures for the Catch-Up Group:

- *Biochemical Testing:* Regular blood tests were conducted to assess the nutritional indicators of the infants;
- Regular evaluations of motor and mental development indexes were performed;
- *Nutritional Intervention*: Appropriate nutritional intervention plans were formulated based on the test results of each infant;
- *Hormone Therapy*: Growth hormone therapies were developed for infants with GH deficiency;
- *Family Training:* During hospitalization, nurses would train family members in skills such as infant massage, exercise activities, kangaroo mother care (KMC), and auditory-visual stimulation;
- Weekly follow-ups via phone were provided to improve the compliance of infants’ family members with the therapy and ensure the accuracy of infants’ data;
- A chat room was established, where scientific knowledge was regularly released and infants’ family members could communicate with ease, helping to alleviate anxiety and depression.

### Evaluation Criteria:

### Physical Development Measurements:

The weight and length of preterm infants were measured by trained nurses, with weight data down to 10 g and height data down to 0.1 cm.^5^ Using the 2006 World Health Organization growth criteria for children aged 0 - 5 years, Z-scores for weight and height and growth rates for SGA infants corrected for age 0 - 24 months were calculated. Z scores = (measured value - mean value for that sex and age) \ (standard deviation for that sex and age), and Z-scores of weight-for-age (WAZ) and height-for-age (HAZ) were calculated, with a standard for catch-up growth defined as ΔZ≥0.67.^6^,^7^ Newborn Apgar Score: The Apgar scores at five minutes after the birth of SGA infants in the two groups were compared, in which the skin color, respiration, heart rate, muscle tone, and response to stimulation were scored, with 0, 1, or 2 points for each item, for a total score of 10 points. A higher score indicated better overall condition.^8^

### Psychomotor and mental development indexes:

Bayley scales of infant development (BISD II) were used to detect the psychological, motor, and behaviors of SGA infants in the two groups for testing, with results expressed as Psychomotor Development Index (PDI) and Mental Development Index (MDI).

### Insulin resistance index:

The levels of fasting blood glucose index (FBG), fasting insulin index (FINS), insulin resistance index (Homa-IR), insulin sensitivity index (Homa-IAI), and islet β cell index (HBCI) were measured at 24 months of corrected age for GSA infants in both groups. The calculation formula: Homa-IR = FBG × FINS/22.5, Homa-IAI = ln [1/(FBG × FINS)], and HBCI = (FINS × 20)/(FBG-3.5).

### Anxiety and depression scores:

Parturients were assessed using the Self-Assessment of Anxiety Scale (SAS) and the Self-Assessment of Depression Scale (SDS), respectively, when infants were at the gestational age of 24 months. SDS scores < 53 and SAS scores < 50 suggested no anxiety and no depression, respectively, with higher scores indicating higher levels of anxiety and depression.

### Statistical analysis:

SPSS 22.0 software was used for data analysis. Count data were expressed as percentages (%), and the χ^2^ test was utilized for comparison. Measurement data were expressed as *χ̅`±S*, and P < 0.05 was considered statistically significant.

## RESULTS

As shown in Table-II and III, no statistically significant differences were observed in body weight and height between the two groups at three months, six months, and nine months of corrected age (P > 0.05). However, the body weight and height in the catch-up group were greater than those in the control group at 12 months, 18 months, and 24 months of corrected age, with statistically significant differences (P < 0.01). Tables-IV and V show no significant differences in WAZ and HAZ values between the two groups at three months, six months, and nine months of corrected age (P > 0.05). However, WAZ and HAZ values in the catch-up group were greater than those in the control group, and the differences were statistically significant (P < 0.01). Table-VI reveals no statistically significant differences in PDI and MDI between the two groups at six months of corrected age (P > 0.05). By contrast, the catch-up group exhibited higher PDI and MDI than the control group at 24 months of corrected age, with statistically significant differences (P < 0.01). As shown in Table-VII, the catch-up group exhibited higher FBG and Homa-IAI indexes and lower FINS, Homa-IR, and HBCI indexes than the control group at 24 months of corrected age, and the differences were statistically significant (P < 0.01). Table-VIII indicates varying degrees of depression in parturients in both groups, and the anxiety and depression in the catch-up group were lower than those in the control group, with statistically significant differences (P < 0.01).

**Table-II:** Comparison of the body weight development index between SGA infants in the 2 groups (N=98,*χ̅*±*S*, g).

Gestational age (month)	Body weight of the catch-up group	Body weight of the control group	t	P
3	5283.64±491.50	5227.19±273.19	0.994	0.322
6	7281.64±328.95	7208.33±375.35	1.454	0.147
9	8369.68±195.56	8362.23±256.02	0.229	0.819
12	9208.65±237.59	8875.50±387.82	7.251	1.641E-11
18	10483.28±465.79	10121.28±421.27	5.706	4.271E-8
24	11864.69±637.52	11588.42±628.81	3.054	0.003

**Table-III:** Comparison of the height development index between SGA infants in the two groups (N=98, *χ̅*±*S*, cm).

Gestational age (month)	Height of the catch-up group	Height of the control group	t	P
3	58.18±1.91	58.41±1.64	-0.903	0.368
6	64.50±2.47	64.24±2.48	0.724	0.470
9	69.87±1.69	69.89±1.00	-0.103	0.918
12	74.42±1.34	73.91±1.08	2.912	0.004
18	81.46±1.07	80.90±0.75	4.206	0.000042
24	87.03±1.70	86.42±1.10	2.976	0.003

**Table-IV:** Comparison of WAZ values between SGA infants in the two groups (N=98, *χ̅*±*S*).

Gestational age (month)	WAZ of the catch-up group	WAZ of the control group	t	P
3	-1.61±1.19	-1.35±2.40	-0.929	0.355
6	-0.94±1.41	-0.90±1.24	-0.200	0.842
9	-0.65±1.67	-0.56±1.51	-0.396	0.692
12	-0.09±1.52	-0.81±1.31	3.564	0.00046
18	-0.10±1.24	-0.96±1.14	5.064	9.498E-7
24	0.07±1.26	-0.36±1.15	2.473	0.014

**Table-V:** Comparison of HAZ values between SGA infants in the two groups (N=98, *χ̅*±*S*).

Gestational age (month)	HAZ of the catch-up group	HAZ of the control group	t	P
3	-1.21±1.05	-1.22±1.10	0.026	0.979
6	-0.81±1.03	-1.01±1.24	1.243	0.216
9	-0.67±1.18	-0.96±1.28	1.651	0.100
12	-0.26±1.18	-0.74±1.35	2.643	0.009
18	0.02±1.26	-0.64±1.34	3.556	0.000474
24	0.01±1.09	-0.53±1.18	3.365	0.001

**Table-VI:** Comparison of PDI and MDI of SGA infants between the two groups (N=98, *χ̅*±*S*).

Gestational age (month)		AGA of the catch-up group	SGA of the control group	t	P
6	PDI	68.07±3.41	66.57±7.12	1.880	0.062
	MDI	69.94±5.62	68.14±7.12	1.959	0.052
24	PDI	89.29±4.35	88.12±3.25	2.121	0.035
	MDI	88.89±4.46	86.69±3.93	3.654	0.000332

**Table-VII:** Comparison of insulin resistance indexes of SGA infants between the two groups (N=98, *χ̅*±*S*).

Index	Catch-up group	Control group	t	P
FINS(μU/mL)	19.40±0.40	22.66±0.88	-33.489	1.134E-67
FBG(mmol/L)	4.41±0.12	4.15±0.08	18.581	7.325E-43
Homa-IR	0.57±0.02	0.63±0.03	-17.558	3.541E-40
Homa-IAI	-2.25±0.03	-2.65±0.04	17.758	1.932E-41
HBCI	65.07±8.80	106.64±13.92	-24.992	8.566E-58

**Table-VIII:** Comparison of emotional scores of parturients between the 2 groups (N=32,*χ̅*±*S*, points).

Group	SDS	SAS
Catch-up	50.86±3.79	46.12±4.96
Control	54.54±3.95	51.38±5.13
*t*	-6.662	-7.293
*P*	2.719E-10	7.521E-12

## DISCUSSION

This study found that the two groups of infants experienced varying degrees of accelerated growth at the age of 0 - 24 months. SGA infants in both groups experienced catch-up growth, with each index in the catch-up group being greater than that in the control group, indicating that scientific and appropriate intervention and nursing were more beneficial for the growth and development of SGA infants. In the field of perinatal medicine, SGA infants may face many challenges in growth and development during the growth stage.^9^,^10^ The causes of SGA are complex and involve multiple factors, including maternal factors, placental and cord factors, genetic factors, fetal and environmental factors, and metabolic diseases.^11^-^13^ The age of 0 - 24 months is a critical period for catch-up growth in SGA infants, during which multifaceted therapeutic approaches mainly include appropriate nutritional and feeding interventions, growth hormone therapy, and neurological interventions.^14^-^16^

By contrast, PDI and MDI were greater in the catch-up group than in the control group at 24 months of corrected age, and the differences were statistically significant (P < 0.01), indicating that early intervention can effectively promote the neurological development of premature infants. Recent studies have found that catch-up growth can promote the occurrence and development of SGA insulin resistance, thereby increasing the risk of obesity, type 2 diabetes, cardiovascular disease and metabolic syndrome.^17^ In the meantime, the catch-up group demonstrated higher FBG and Homa-IAI indexes and lower FINS, Homa-IR, and HBCI indexes than the control group at 24 months of corrected age, with statistically significant differences (P< 0.01). With the lower-than-average value of indexes in infants, the family members of the control group lack professional cognition and nursing knowledge and are prone to providing excessive nutritional supplementation, resulting in premature infants with overnutrition and ultimately leading to insulin resistance.

In the control group, the side effects of catch-up growth were prevented through regular tests for various indexes, moderate adjustment of nutritional structure, and appropriate nutritional intervention. At present, domestic and foreign studies on SGA mainly focus on nutrition and feeding promotion.^18^,^19^ However, a single promotion method may lead to SGA obesity, and then lead to a series of health problems, such as developmental delay and insulin resistance, which will adversely affect the long-term health of SGA.^20^ In this study, multiple promotion methods were comprehensively evaluated to avoid the excessive influence of a single method on other aspects of SGA growth and development, and to construct a scientific and effective catch-up plan. It has been reported that the growth pattern of SGA varies in different months, from the regulation of nutritional metabolism in the fetal period, to the growth-promoting effect of pituitary GH system after 6 months, and to the auxin axis regulation from December to 24 months.^21^ It is crucial to provide timely and effective treatment for SGA infants, and the efficacy of subsequent treatments will be significantly compromised if missing the optimal treatment window.

### Limitations

This study focused on assessing growth and development in infants aged 0 - 24 months. However, the existing research is insufficient regarding the potential health risks for SGA infants in adulthood. To ensure the comprehensiveness and depth of the study, long-term follow-up studies are necessary to continuously monitor the growth and development of these infants. Additionally, the specific effects of catch-up growth plan on neurological and physical development for SGA infants in adulthood should be further explored, thereby compensating for the limitations of current short-term studies.

## CONCLUSIONS

Appropriate intervention programs can effectively promote the growth and development of SGA and improve its prognosis. On the premise of ensuring that the growth and development indicators of SGA are basically consistent with the effect of a single study, it also increased the attention and training for the families of children with SGA, avoided the possible side effects of improper intervention measures, made the intervention method more scientific and comprehensive, and provided a better treatment plan for treatment. It has important clinical significance to improve the future life quality and health level of SGA.

### Authors’ Contributions:

**LS** and **JG:** Carried out the studies and are responsible and accountable for the accuracy or integrity of the work.

**WD** and **SZ:** Literature search, participated in its design and drafted the article.

**JL:** Collected the data and performed the analysis. Critical Review.

All authors have read and approved the final manuscript.
